# Simulation of Pulse-Echo Radar for Vehicle Control and SLAM

**DOI:** 10.3390/s21020523

**Published:** 2021-01-13

**Authors:** Girmi Schouten, Wouter Jansen, Jan Steckel

**Affiliations:** 1 CoSys-Lab, Faculty of Applied Engineering, University of Antwerp, 2020 Antwerpen, Belgium; wouter.jansen@uantwerpen.be (W.J.); jan.steckel@uantwerpen.be (J.S.); 2 Flanders Make Strategic Research Centre, 3920 Lommel, Belgium

**Keywords:** radar, simulation, vehicle control, SLAM, biologically-inspired

## Abstract

Pulse-echo sensing is the driving principle behind biological echolocation as well as biologically-inspired sonar and radar sensors. In biological echolocation, a single emitter sends a self-generated pulse into the environment which reflects off objects. A fraction of these reflections are captured by two receivers as echoes, from which information about the objects, such as their position in 3D space, can be deduced by means of timing, intensity and spectral analysis. This is opposed to frequency-modulated continuous-wave radar, which analyses the shift in frequency of the returning signal to determine distance, and requires an array of antenna to obtain directional information. In this work, we present a novel simulator which can generate synthetic pulse-echo measurements for a simulated sensor in a virtual environment. The simulation is implemented by replicating the relevant physical processes underlying the pulse-echo sensing modality, while achieving high performance at update rates above 50 Hz. The system is built to perform design space exploration of sensor hardware and software, with the goals of rapid prototyping and preliminary safety testing in mind. We demonstrate the validity of the simulator by replicating real-world experiments from previous work. In the first case, a subsumption architecture vehicle controller is set to navigate an unknown environment using the virtual sensor. We see the same trajectory pattern emerge in the simulated environment rebuilt from the real experiment, as well as similar activation times for the high-priority behaviors (±1.9%), and low-priority behaviors (±0.2%). In a second experiment, the simulated signals are used as input to a biologically-inspired direct simultaneous mapping and localization (SLAM) algorithm. Using only path integration, 83% of the positional errors are larger than 10 m, while for the SLAM algorithm 95% of the errors are smaller than 3.2 m. Additionally, we perform design space exploration using the simulator. By creating a synthetic radiation pattern with increased spatiospectral variance, we are able to reduce the average localization error of the system by 11%. From these results, we conclude that the simulation is sufficiently accurate to be of use in developing vehicle controllers and SLAM algorithms for pulse-echo radar sensors.

## 1. Introduction

Echolocation is a sensing method used by some species of bats and dolphins to navigate their surroundings, detect obstacles, and classify prey [1]. They accomplish this through biological sonar; they emit ultrasonic signals from their vocals tract, which reflects off objects in the environment and are received as echoes by their ears. From these echoes, they can determine the location of the reflector, as well as other properties such as size, velocity and even texture [2,3]. Although sonar is generally considered a primitive sensing modality in robotics, these animals refute this fact by virtue of their navigation, foraging and hunting abilities based on their sonar sense. Thus, echolocation can serve as an inspiration for technological solutions that require unconventional sensing methods, while minimizing hardware and computational complexity. It has already been shown that acoustic sensing can approach or even rival visual approaches, especially when combined with recent advances in machine learning [4,5]. As technology moves to increasing levels of automation and autonomy, the requirements for such systems increase, including the circumstances in which they are expected to operate. As no single sensor is able to provide reliable sensing under all circumstances, systems need to be equipped with multiple sensors that complement each other in various scenarios [6].

Prior work has shown how biological echolocation can be mimicked by electronic pulse-echo sonar to create robust, lightweight, and economical sensors [7]. These sensors are able to perform simultaneous localization and mapping (SLAM), and autonomous navigation on robotic platforms in various settings [7,8]. Further study of biological sonar shows that there is also a potential for other applications such as target tracking and interception, as well as increased precision by means of adaptive echolocation [9]. In addition to this biologically-inspired sonar senor, we have also developed a biologically-inspired radar sensor [10]. It operates according to the same principles of biological echolocation, but uses electromagnetic waves instead of acoustic waves. In previous work, we have demonstrated that this sensor has capabilities similar to its sonar counterpart [11,12], with the advantage of improved performance in certain circumstances such as high humidity and high-temperature environments [13,14].

The biologically-inspired radar sensor has proven to be effective in real-world applications, yet there is still room for further development. One promising area for improvement is the radiation pattern of the antennas. The high spatiospectral variance could allow the system to better locate reflectors in 3D space, which translates to better performance in autonomous navigation and place recognition [15]. However, while changes to the signal processing software can be carried out with relative ease, modifications to the hardware are more time and resource-consuming. To overcome this obstacle, this work presents a newly developed pulse-echo radar simulator. The goal of this simulator is to facilitate design space exploration by narrowing down promising prototypes in simulation before implementing them. In addition, this simulator also allows for testing of experimental hardware and software in the safety of a virtual environment, as well as in a multitude of settings which might otherwise not be accessible, e.g., an industrial factory hall or a public road. Lastly, it is also possible to integrate other sensing modalities, such as lidar and camera’s into the simulator to perform sensor comparison or to implement sensor fusion [16]. Similar efforts have been done for frequency-modulated continuous-wave (FMCW) radar where realistic radar observations are generated from elevation maps of simulated environments [17]. Our simulator works by approximating signal propagation by ray tracing. These rays mimic the interactions of a signal with the environment and undergo effects such as transmission, absorption, reflection. Although this approach does not encompass all wave effects, it provides a sufficient level of realism for the purpose of simulating our type of sensor [18]. Ray tracing searches for reflection paths from the emitter to the receiver, given an environmental model and the current sensor pose. Is then possible to synthesize a waveform with echoes for a given sensor configuration, based on the path information of the reflections. Our simulator is built on top of the Unreal Engine, a game engine which can simulate complex environments populated with detailed objects and advanced physics interactions [19]. In addition, we use the Microsoft AirSim plugin, which provides a collection of virtual vehicles and exposes several interfaces to facilitate communication with the engine [16]. Leveraging these existing tools increases the capabilities, flexibility and ease of use our the final product. MATLAB is used as the signal processing component of the simulator, communicating with the Unreal engine to retrieve the reflector point cloud from which to generate the synthetic waveform. Although other ray-tracing approaches to high-frequency radar propagation exist, most of them use custom implementations instead of common and readily available software suites [20].

The rest of the paper is structured as follows. Section 2 gives an overview of the principles at play in biological echolocation and pulse-echo radar systems. These principles are used to guide the development of the simulator. Section 3 explains the implementation of the radar simulation, detailing the capabilities as well as limitations of our approach. Section 4 demonstrates how the simulator can be used to carry out autonomous control of robotic platforms. To validate the performance of the simulator, we replicate real-world experiments from previous research. Section 5 presents the results of SLAM experiments in simulation and again compares them to their real-world counterparts. Lastly, in Section 6 we perform design space exploration by altering the radiation pattern of the sensor in simulation to improve SLAM performance.

## 2. Pulse-Echo Radar Principles

The purpose of a simulation is to replicate a real-world process or phenomenon. In this work, our goal is to simulate the output of our biologically-inspired pulse-echo radar sensor, given an environment, a sensor model and the location of the sensor within this environment. The simulator then outputs a set of signals in which echoes of objects in the environment are present. To explain how the simulated sensor is implemented, we first give an overview of the aspects of the physical sensor which are relevant to the application of the simulator.

**Figure 1:**
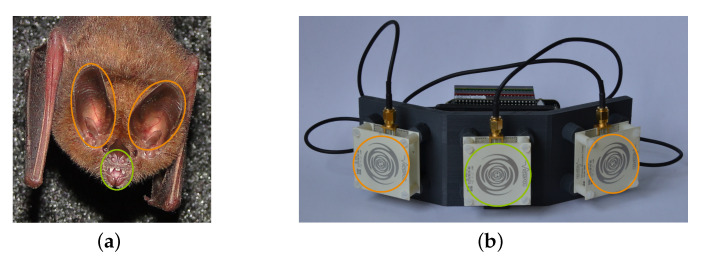
Comparison of the sensing mechanisms in biological echolocation and electronic echolocation. In both images, the emitter is marked green, and the receivers are marked orange. (**a**) The common big-eared bat (*Micronycteris microtis*). (**b**) A modified SensorLogic Ancho pulse-echo radar sensor.

Echolocation works by emitting a signal pulse into the environment and picking up any echoes that are caused by reflecting objects in this environment. By analyzing these echoes, it is possible to deduce the relative distance and direction to each reflector, as well as other properties. In biological echolocation, the animal’s vocal tract serves as the emitter and its two ears as receivers, as indicated in Figure 1a. Its brain does the processing to convert the acoustic signals to a set of object locations which can be interpreted by the animal. In our radar sensor, this process is mimicked by using an emitting antenna and two receiving antennas, which are connected to a processing unit, as seen in Figure 1b, which in turn converts the radar signals to a digital representation of object locations. The hardware platform we use in our research is the SensorLogic Ancho Radar Development Kit [21]. It consists of a Novelda XeThru X2 radar system on a chip (SoC), a BeagleBone Black back-end and sinusoidal antennas.

To perform a measurement, the radar SoC generates an electromagnetic waveform, in this case a Gaussian-modulated sinusoid pulse with a center frequency of 5.5 GHz and bandwidth of 3 GHz, corresponding to wavelengths of 4 to 7.5 cm. The pulse then travels along the transmission lines to the antenna. Once it reaches the antenna, it is propagated into free space, with it’s spatiospectral profile determined by the characteristic radiation pattern of the antenna. From there, on the electromagnetic wave travels through space until it either dissipates due to attenuation or interacts with an object. Several factors contribute to the attenuation of a wave, the most fundamental being free-space path loss and antenna gains. These are captured in the Friis transmission formula. Additionally, signal attenuation is also influenced by the propagation medium according to the atmospheric attenuation. It includes absorption by gasses present in the atmosphere, such as oxygen and hydrogen, as well as airborne particles such as rain, fog, dust [13]. We expand the Friis equation to include this phenomenon;

(1) $$P_{r}=P_{e}\;\cdot \;G_{e}\left(\psi ,\lambda \right)\;\cdot \;G_{r}\left(\psi ,\lambda \right)\;\cdot \;{\left(\frac{\lambda }{4\pi d_{l}}\right)}^{2}\;\cdot \;Q\left(\lambda ,d_{l}\right)$$

with

$P_{r}$: the power of the received wave;
$P_{e}$: the power of the emitted wave;
$G_{e}\left(\psi ,\lambda \right)$: the gain of the emitting antenna;
$G_{r}\left(\psi ,\lambda \right)$: the gain of the receiving antenna;
λ: the wavelength;
ψ: the direction of incidence, composed of azimuth and elevation components;
$d_{l}$: the length of the path between the emitter and receiver.
*Q*: the atmospheric attenuation.

An unobstructed wave continues to propagate until it dissipates, meaning its power has diminished to a level where it becomes too weak to detect for all practical purposes. However, when a wave collides with an object, it can interact with the object in several ways:

- Transmission: the wave passes through the object. This can include refraction, in which the direction of the wave is altered. Depending on the surface structure and frequency of the wave, transmission can either be translucent or transparent;
- Absorption: the wave is taken in by the object and dissipated as heat;
- Reflection: the wave is reemitted from the object’s surface. Depending on the structure of the surface and frequency of the wave, reflection can either be specular or diffuse;
- Diffraction: the wave is bent or distorted when encountering an edge, slit, or aperture.

The majority of waves travel through the environment until dissipated or absorbed. However, a small minority reaches the sensor after one or more reflections. Once such an echo reaches the receiving antenna, it again is filtered by the radiation pattern of the antenna according to the principle of reciprocity. After reception, the pulse travels along the transmission line to the radar SoC, where it is digitized and processed further by the signal processing software.

The way in which echoes exhibit themselves in a measured waveform depends on several phenomena. In active sensing, we consider the moment of emission as the start of the measurement. Thus, the location of an echo in a time signal is determined by the distance to the reflector and the signal velocity [22];

(2) $$t_{e}=\frac{2\;\cdot \;d_{r}}{v_{s}},$$

with

$t_{e}$: the timing of the echo;
$d_{r}$: the distance to the reflector;
$v_{s}$: the signal velocity. For radar, it is the speed of light in air.

Technically, the timing is determined by the distance from the emitter to the reflector and then from the reflector to the receiver. However, as the emitter and receiver are co-located in most echolocation systems, this distance becomes equal to twice the distance from the sensor to the reflector. Another influence is that, in a binaural system such as the mammalian auditory system, echoes are received separately in each ear. Because the ears are located at different positions on the head, there are minute differences in arrival time between the left and right channel [23]. This difference is called the interaural time difference (ITD);

(3) $$\Delta t_{e}=\frac{d_{c}\;\cdot \;sin\left(\theta_{i}\right)}{v_{s}}$$

with

$\Delta t_{e}$: the timing difference between channels;
$d_{c}$: distance between the channels’ receivers;
$\theta_{i}$: angle of incidence of the echo in the plane of the receivers;

A related phenomenon is the difference in intensity with which an echo is received at each channel. In biological systems, it is known as the interaural intensity difference (IID), Because the ears are located on opposing sides of the head, an incident echo might arrive at each ear at a different angle, and can be partially blocked by the head. This means the energy of an echo will differ depending on its angle of incidence. This effect depends on the physical arrangement of the head, such as its size and the relative placement of the ears. Therefore, no general equation is available to predict its influence on an echo, and it has to be empirically determined by measurement [24]. In radar, these effects translate to the relative positions of the receiver antennas as well as the properties of the material in-between them.

In addition to the previous phenomena, signals are also filtered during emission and reception. This filtering is a consequence of the physical interaction between the signal, and the emitter and receiver, and depends on the direction of incidence, frequency of the signal, and physical properties of the antennas. In passive sensing, such as human hearing, this filtering is characterized by a head-related transfer function (HRTF) [25]. It describes the response of the receiver when picking up a signal from a specific direction in space;

(4) $$S^{\prime }\left(f,\psi \right)=H\left(f,\psi \right)\;\cdot \;S\left(f\right)$$

with

$S^{\prime }\left(f,\psi \right)$: the received signal spectrum;
H(f,ψ): the HRTF of the receiver;
S(f): the spectrum of the incoming signal.

In the time domain, this process is represented by the convolution of the head-related impulse response (HRIR) with the incoming signal. The result is a superposition of the original signal with the reflections and shadowing caused by the external ear;

(5) $$s^{\prime }\left(t,\psi \right)=h\left(t,\psi \right)*s\left(t\right)$$

with

$s^{\prime }\left(t,\psi \right)$: the received signal;
H(t,ψ): the HRIR of the receiver;
s(t): the incoming signal.

Because the auditory system is binaural, an HRTF or HRIR of both ears is required to fully describe the characteristics of the system. Furthermore, as echolocation is an active sensory method, it probes the environment using self-generated signals. This involves the sensor applying filtering to the signal during emission, as well as during reception. Therefore, an extra factor is needed to take into account this additional filtering;

(6) $$S^{\prime }\left(f,\psi \right)=H_{e}\left(f,\psi \right)\;\cdot \;S\left(f\right)\;\cdot \;H_{r}\left(f,\psi \right)$$

with

$H_{e}\left(f,\psi \right)$: the HRTF of the emitter;
$H_{r}\left(f,\psi \right)$: the HRTF of the receiver.

Because the emitter and receiver are tightly coupled in echolocation systems, and sometimes cannot even be characterized separately, their combined effects on an echo are commonly captured in the echo-related transfer function (ERTF) E [26];

(7) $$\begin{array}{cc} E(f,\psi ) & =H_{e}\left(f,\psi \right)\;\cdot \;H_{r}\left(f,\psi \right) \end{array}$$

(8) $$\begin{array}{cc} S^{\prime }\left(f,\psi \right) & =S(f)\;\cdot \;E(f,\psi ). \end{array}$$

The ERTF of our biologically-inspired radar has to be determined experimentally, as we have no model available from which its transfer function can be directly derived. To determine the ERTF, the sensor is mounted on top of a pan-tilt unit and placed in front of a radar retroreflector in an electromagnetic anechoic chamber. The sensor is oriented along equally-spaced directions on a sphere, and for each direction, a measurement is made. These measurements are then transformed to the frequency domain and divided by the spectrum of the emitted pulse to obtain the ERTF of the sensor. It is also possible to generate synthetic ERTFs by projecting a two-dimensional intensity function onto a sphere surface, which allows for testing of a variety of, possibly hypothetical, antenna models without the need to build physical prototypes.

In the next section, we will show how the above principles are integrated into our simulator. For further information on how these phenomena are used to determine the exact location of reflectors in the environment, we refer to [10], as this it is not the focus of the current work.

## 3. Pulse-Echo Radar Simulation

This section describes how the process of pulse-echo radar is implemented in our simulator. Simulation is always an approximation of reality, either because of imperfections in the understanding of the phenomena involved, or because of practical limitations on computational power and precision. Therefore, we implement only the underlying processes which are relevant to our application and try to mitigate the shortcomings of the approximation as much as possible.

There are two major approaches to simulate signal propagation through space; wave-based techniques evaluate the wave equation numerically at each point in space to simulate how a signal propagates through the environment [27]. Ray-based techniques, also known as geometrical approaches, approximate wavefronts by bundles of energy packets moving through space along distinct rays [28]. Ideally, one would use a wave-based approach, as it encompasses all phenomena which relate to signal propagation. However, the required mesh resolution scales $O(n^{3})$ with the signal frequency while the simulation time step scales $O(n^{3}\log n^{3})$, making it infeasible for high-frequency applications in large-scale environments such as ours [28]. As typically 5 elements per wavelength are required, this would result in a mesh of 1,000,000 elements per cubic meter at a frequency of 6 GHz.

On the other hand, ray-based approaches are assumed valid in situations with a large Helmholtz number H;

(9) $$H=\frac{2\pi L}{\lambda },\;L\gg \lambda$$

with

*L*: the characteristic length of the geometry;
λ: the wavelength of the signal.

**Figure 2:**
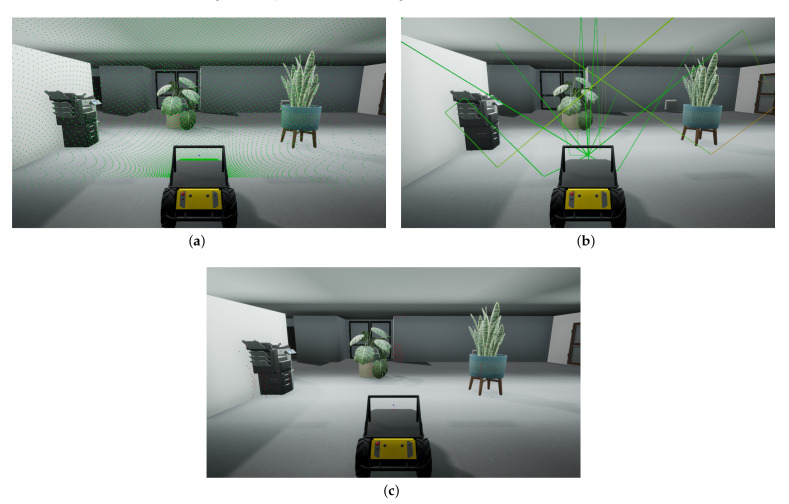
Overview of the simulation steps in the Unreal engine. (**a**) The impact locations of the initial rays cast into the environment (green dots). (**b**) The paths of selected rays as they propagate through the environment (colored lines). (**c**) The locations of reflected rays that have reached the sensor (red dots).

This condition holds for high-frequency signals in large-scale environments. It is applicable to our scenario with signal wavelengths ranging from 4.5 to 7.5 cm, in rooms with wall surfaces larger than 1 m${}^{2}$ and office decorations larger than 30 cm, as can be seen in Figure 2. The computational cost of this technique is independent of signal frequency, but instead scales linearly with the spatial resolution and the lower bound of the detectable signal. There are limitations to the ray-based approach, which we will briefly discuss here. First, rays are only cast in a limited amount of discrete directions, and thus do not cover the entire surroundings within the line of sight. This means the environment is spatially undersampled, and high frequency geometric features are not captured. Since rays are cast radially outward, this effect is larger for objects farther away. Therefore, a trade-off has to be made between speed and accuracy, depending on the requirements of the simulation and the computational power available. Second, ray tracing does not inherently account for any diffraction effects. However, as the wavelength of the signal is much smaller than the relevant geometric features in our case, these effects are nearly imperceptible and can therefore be disregarded. Lastly, diffuse reflections cause signals to be scattered in multiple directions. This can be replicated by splitting a single ray into multiple rays upon reflection. However, this increases the number of reflecting rays exponentially, and therefore adds a large computational burden. Because spectral reflections are the most relevant to our application, we do not perform diffuse reflections, as this would increase simulation time significantly without benefiting the end-result.

The pulse-echo radar simulation stack consists of two main components; signal propagation through the virtual environment, and signal waveform generation, which we will discuss in the rest of this section. The first is handled by the Unreal engine, the latter by MATLAB.

### 3.1. Signal Propagation

To generate an artificial measurement, it is necessary to determine which echoes will be received by the sensor given its current pose and its surroundings. This is done by ray tracing, which reproduces the way a signal propagates through free space and how interacts with objects along its path. If the reflected signal passed through the sensor’s receptive field, it produces an echo waveform in the resulting measurement.

#### 3.1.1. Sample Directions

To perform a measurement, we first determine the directions in which rays will be cast. To sample the environment as uniformly as possible, these directions are calculated by using the recursive zonal equal area sphere partitioning algorithm [29,30]. It achieves this by partitioning the sphere into a number of collars and subdividing these collars into sections. Taking the center of each section then results in a set of equally spaced points on the surface of the sphere. The output of this algorithm can be adjusted by two parameters which determine the total number of rays and the opening angle of the ray bundle. Figure 2a shows the direction distribution of 10,000 rays over an opening angle of 180° as they are cast into the environment and strike the first surface in their line of sight.

#### 3.1.2. Ray Casting

For a single measurement, rays are cast along all sample directions. Each ray has two properties associated with it; its current attenuation $A_{r}$ and distance traveled $d_{r}$. Once a ray is emitted, it travels until it collides with an object or dissipates. Dissipation is determined by the attenuation limit $\hat{A}$, specified by the user as the lowest detectable signal level. During propagation, a ray attenuates due to two difference phenomena; free-space path loss and atmospheric attenuation. Free-space path loss is implemented according to the second to last term of Eguation (1), using the distanced traveled so far, and the wavelength of the signal. Atmospheric attenuation, as denoted in the last term of Equation (1), is computed based on the currently active weather in the simulation, which can be dynamically changed. Our implementation of the simulator allows the user to define a distinct attenuation factor for each type of weather, e.g., rain, snow, fog, dust [13]. If at any point during the simulation the accumulated attenuation of a ray exceeds the attenuation limit, the ray is discarded. To improved performance, we include a user-defined parameter to limit the maximum length of the path of the signal. It should be proportional to the duration of the measurement and the signal speed.

#### 3.1.3. Collision and Reflection

If the path of a ray results in a collision, the direction of the ray is altered according to the law of specular reflection;

(10) $$\vec{d_{r}}=\vec{d_{i}}-2\;\cdot \;\left(\vec{d_{i}}\cdot \vec{d_{n}}\right)\;\cdot \;\vec{d_{n}}$$

with

$\vec{d_{i}}$: the incident direction, from the ray’s origin to the impact point;
$\vec{d_{n}}$: the direction of the surface normal, retrieved from the object mesh;
$\vec{d_{r}}$: the resulting reflection direction;

Each collision absorbs part of the energy of the signal, based on the material properties of the colliding object. This absorption is represented by an increase in the attenuation of the ray, and can be defined by the user for different object materials, such as metal, concrete, and wood [31]. Next, a new ray is cast from the impact location along the direction of reflection, and the process of attenuation and reflection continues. Additionally, each time a reflection occurs, we verify that the remaining length of the signal path is sufficient to return to the sensor. If not, the ray is discarded, as it will not be able to generate an echo. Furthermore, we also include a parameter to control the maximum order of reflections, to limit the computational cost of the simulation. An example of the paths rays traces as they propagate through the environment and reflect off of surfaces is shown in Figure 2b.

#### 3.1.4. Reception

After reflection, we determine if the outgoing ray intersects the sensor. This is done by checking if the reflection’s direction lies within the user-defined opening angle of the sensor.

(11) $$\begin{array}{c} \theta_{r}=\arccos\left(\frac{-\vec{d_{s}}\cdot \vec{d_{r}}}{\vec{d_{s}}\;\cdot \;\vec{d_{r}}}\right) \end{array}$$

(12) $$\begin{array}{c} R=\left\{\begin{array}{cc} \mathrm{true}, & \mathrm{if}\;\theta_{r}\leq \theta_{s}.\\ \mathrm{false}, & \mathrm{otherwise}. \end{array}\right. \end{array}$$

with

$\vec{d_{s}}$: the heading of the sensor
$\vec{d_{r}}$: the direction of the reflected ray
$\theta_{r}$: the reception angle
$\theta_{s}$: opening angle of the sensor
*R*: reception conditional

If the reception angle is smaller than the opening angle of the sensor, a ray is cast from the reflection site to the sensor, to check if there is a clear line of sight between them. If this is the case, an echo occurs and the origin of the reflection is registered. In addition, ray collisions with objects in the near-field of the emitter are always considered reflections, as they do not adhere to the same the laws of reflection as far-field signals [32]. For this purpose we consider the near field as the region closer than the start of the far-field, thus any distances d<2λ. Figure 2b shows the final set of locations from which an echo was received by the sensor. These locations contribute to the echoes which will be present in the measured signal.

The performance of the ray-tracing pipeline for 25,000 rays with a maximum distance op 20 m and reflection depth of 5 delivers an update rate of 50 Hz on an AMD Ryzen 3700X CPU. This is sufficiently high for real-time execution of the simulation, which enables the use of user input or other types of real-time interaction. Additionally, it is possible to further increase the spatial accuracy of the simulation at the cost of decreased performance, as the update rate scales inversely with the number of rays.

### 3.2. Signal Generation

Once all rays have dissipated and all reflections are registered, the point cloud of reflector locations is sent to MATLAB for further processing and signal generation. Each point includes the total ray length before reaching the sensor and the total attenuation it accumulated along the way.

#### 3.2.1. Emitted Waveform

The first step in this process is to generate the emitted signal waveform, which only has to be done once for a specific sensor configuration. The signal can be chosen freely, but in this case we use one resembling that of the physical pulse-echo radar system, namely a Gaussian-modulated sinusoid;

(13) $$s_{p}\left(t\right)=\sin\left(2\pi \;\cdot \;f_{c}\;\cdot \;t\right)\;\cdot \;w_{g}\left(t\right)$$

with

$f_{c}$: the center frequency of the pulse
$w_{g}$: the Gaussian window function
$s_{p}$: the pulse waveform at time *t*

where the Gaussian window function is defined as;

(14) $$w_{g}\left(t\right)=\exp\left(-\frac{1}{2}\;\cdot \;\frac{{\left(t-∆t\right)}^{2}}{\sigma^{2}}\right)$$

with

∆t: the time shift of the window
σ: the width of the window, which determines the bandwidth

**Figure 3:**
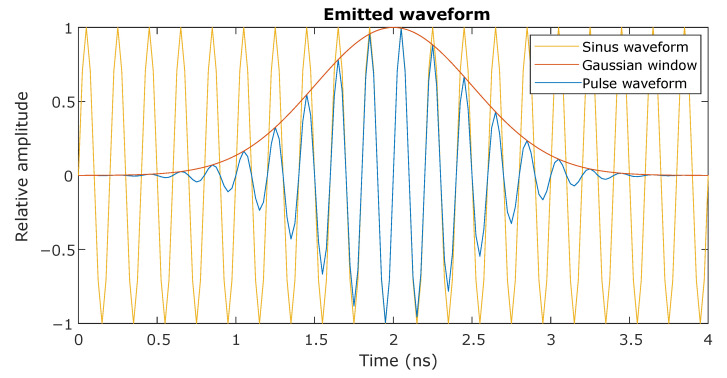
The emitted signal used for simulation the radar sensor. The sinusoid carrier (yellow) is modulated with a Gaussian window filter (orange) to produce a pulse signal (blue).

The resulting pulse and its components are shown in Figure 3.

#### 3.2.2. Point Cloud Processing

**Figure 4:**
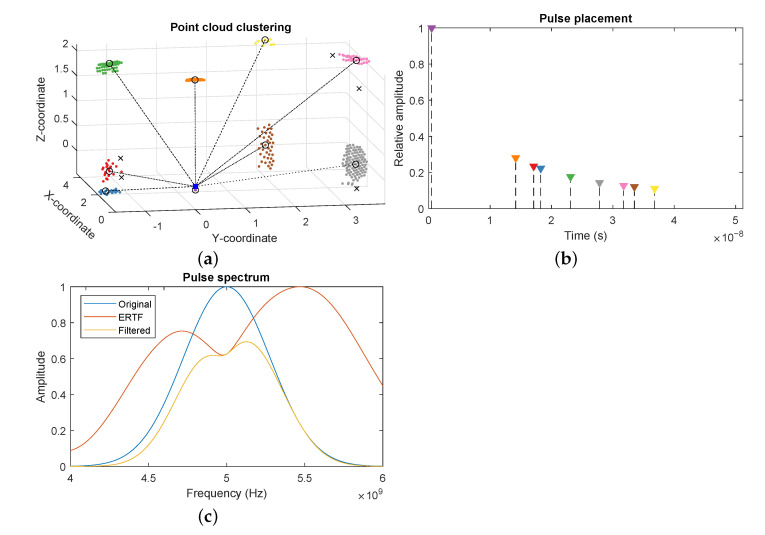
Steps of the reflector point cloud processing in MATLAB. (**a**) The point cloud received from the Unreal engine and clustered by the density-based spatial clustering of applications with noise (DBSCAN) algorithm. Clusters are color-coded, cluster centers are marked with circles, outliers are represented by crosses. The sensor location is draw as a blue square. (**b**) Pulse placement and amplitude in the generated signal according to the reflector location and ray attenuation. (**c**) Filtering of a pulse by the emitter and receiver for a specific incoming direction.

Due to the dense sampling of the ray casting process, some surfaces might return a large set of closely spaced reflector locations. This increases the computational costs of the waveform generation and can introduce artifacts due to the overlap of large numbers of echoes. Therefore, we cluster the global point cloud using the density-based spatial clustering of applications with noise (DBSCAN) algorithm [33]. This algorithm takes two parameters; the cluster size, which determines the maximum distance between points in a cluster. A larger cluster size means a more sparse point cloud, but possibly clustering together points from different reflection paths. The second parameter determines the minimum number of points per cluster. Due to the discrete nature of the ray-tracing method and the tessellation of the environment, available reflection paths might change rapidly between time steps, causing flickering in the point cloud. By increasing the minimum amount of points required to form a cluster, the averaging effect counters this flickering, resulting in a more stable point cloud. For each cluster, we use the centroid as the new location, and average the distance and attenuation of all points in the cluster. This process is visualized in Figure 4a, showing the original point cloud divided into the detected clusters, and the clusters centers which form the reduced point cloud. The outliers are dropped as they are most likely unstable reflection paths which would introduce artifact reflection into the measurement.

#### 3.2.3. Signal Filtering and Sequencing

Next, an echo is added to the signal for each point in the point cloud. Starting from the emitted waveform, each echo is filtered according to the ERTF of the receiver, depending on the reflection’s angle of incidence. To achieve this, we match the incoming direction to the closest direction in the ERTF, and apply the specific spectral gains for that direction to the spectrum of the pulse, according to Equation (8). An example of this filtering can be seen in Figure 4c, where we show the spectrum of an incoming pulse being filtered by the ERTF for an arbitrary direction.

We then calculate the pulse amplitude from its accumulated attenuation;

(15) $$a=10^{\frac{A}{20}}$$

with

*a*: the amplitude of the pulse
*A*: the attenuation of the echo

and its position in the signal according to Equation (2);

(16) $$n=｢t_{e}\;\cdot \;f_{s}｣$$

with

$f_{s}$: the sample rate of the sensor
*n*: the sample location of the pulse

These calculation are depicted in Figure 4b, where the location of each line represents the placement of the echo of the corresponding cluster, and the height represents the amplitude. Each filtered pulse is then superimposed onto the accumulated signal. When all echoes are processed, additive white Gaussian noise is applied to replicate the internal system noise;

(17) $$\begin{array}{c} s\left[n,\cdots ,n+l_{p}\right]=a\;\cdot \;s_{p} \end{array}$$

(18) $$\begin{array}{c} s=s+Z \end{array}$$

with

*s*: the simulated signal
$l_{p}$: the length of the emitted pulse
Z: the zero-mean, normal distributed noise vector

**Figure 5:**
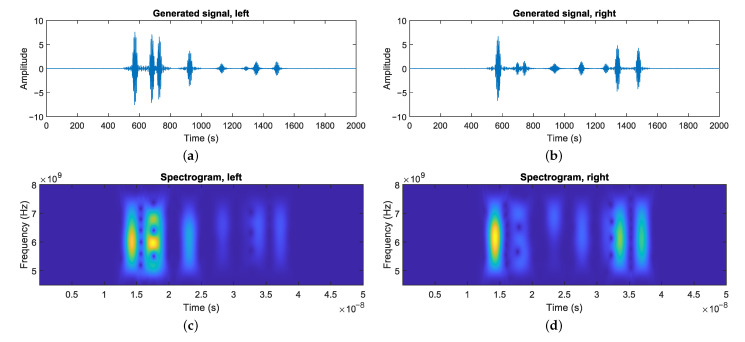
A complete measurement produced by the simulator. (**a**,**b**) The waveform measurements using the simulated radar sensor in a virtual environment for the left and right channel. The initial pulse is omitted for improved visibility. (**c**,**d**) The spectrogram representation of the signals.

The final result of this process can be seen in Figure 5a. It shows the signal waveform generated by a measurement from the simulated sensor in the virtual environment.

## 4. Autonomous Navigation

To validate the performance of our simulation implementation, we set out to replicate the results of a real-world experiment conducted using the pulse-echo radar sensor in previous work, which allows us to directly compare our simulation to a ground truth [11]. Here, we mounted the radar sensor on a robotic vehicle and used it as the sole input for a behavior-based control algorithm which was set to traverse the environment without collision.

### 4.1. Subsumption Architecture

In this section, we provide a brief overview of behavior-based control, but refer to [11] for a more detailed explanation. Subsumption architecture is an implementation of behavior-based control in which the overall actions of a robotic system are determined by the decisions and interactions of a set of distinct behaviors. Each behavior has a priority and set of conditions associated with it, and is responsible for controlling the robot in a specific scenario. At each time step, all behaviors are given the current measurements from the sensors, from which each behavior determines if its activation conditions are fulfilled. An arbitrator then selects the highest priority behavior and gives it control over the robot by passing its velocity outputs to the controller.

#### 4.1.1. Collision Detection

Collision detection (CD) is the highest priority behavior in this setup. It is responsible for detecting when the vehicle has collided with an object and tries to recover from this scenario by returning the vehicle to a state from which another behavior can take over.

#### 4.1.2. Collision Avoidance

Collision avoidance (CA) tries to prevent the collisions by halting the vehicle and turning away when an object is detected in close proximity. Once the vehicle has a clear path ahead, the behavior will relinquish control to the other behaviors to continue on its route.

#### 4.1.3. Obstacle Avoidance

Obstacle avoidance (OA) takes into account upcoming obstacles on the vehicle’s path to steer the vehicle around them. It does this while maintaining forward velocity, resulting in a smooth trajectory. This behavior is also capable of guiding the vehicle through narrow passages such as doorways by balancing reflection from symmetrically placed reflectors.

#### 4.1.4. Corridor Following

Corridor following (CF) navigates the vehicle through its surroundings in absence of any approaching obstacles. It does this by balancing the change of energy in each channel, similar to the principle of optical flow in mammal or insect vision [34]. The dynamics of this behavior cause the vehicle to center itself between large-scale features such as walls, leading it to follow stable paths through the environment.

#### 4.1.5. Straight Drive

Straight drive (SD) is the lowest priority behavior in this setup and will only take over when no other behavior is active. It simply continues the vehicle along its current trajectory until it encounters a scenario in which another behavior takes priority.

### 4.2. Experimental Results

In the original real-world experiment, we used the SensorLogic Ancho Radar mentioned in Section 2 mounted on a Pioneer P3DX vehicle. To reproduce this experiment, the simulated sensor was calibrated as closely as possible to the original sensor, by utilizing the sensor ERTF from a recording and by replicating the emitted signal as closely as possible. Additionally, the simulated environment was rebuild from the original 2D map. This was done by extruding the walls in height, and adding the floor and ceiling. Other objects and environmental features were manually added.

**Figure 6:**
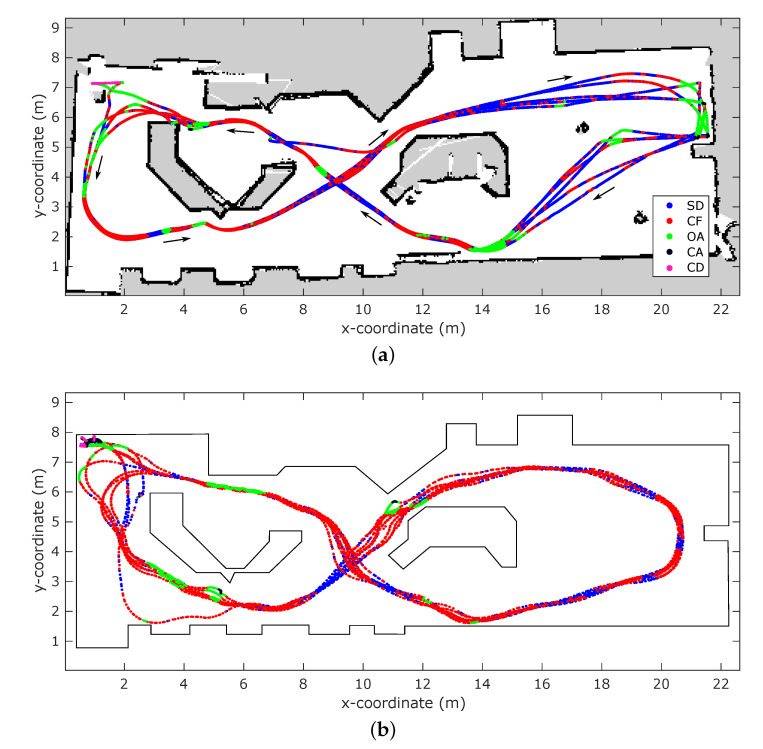
Comparison of the behavior control results of a real-world experiment versus a simulated experiment. Each dot represents a pose of the vehicle. Dots are colored according to the active behavior at that moment. (**a**) Active behaviors for the trajectory of vehicle in a real-world experiment from previous work. (**b**) Active behaviors for the trajectory of the simulated vehicle in the virtual environment.

A comparison of the resulting trajectories and behavior activity is presented in Figure 6. It shows that the behavioral control algorithm using the simulated radar in a virtual environment produces stables paths, similar to the real-world experiments conducted in previous work. The overall structure and stability of the paths are comparable; the eight-shape of the trajectory with overlapping paths on consecutive passes through the environment. It can be noted that are differences in sections of the trajectories, specifically, in the upper left and right corners of the map, where either the real or simulated vehicle gets stuck and must turn around to continue. These partial deviations might be the result of imperfect modeling of the environment or sensor, causing different dynamics.

In the simulated experiments, CD was active for 0.9% of the trajectory, CA for 3.1%, OA for 9.7%, CF for 58.2% and SD for 28.1%. For the real-world experiment, these numbers were 0.4%, 0.8%, 12.7%, 34.2% and 51.9% respectively. For the three highest-priority behaviors the activation times are similar to those in the real-world experiment, with an average deviation in activation time of ±1.9%. The largest differences can be found in the activity of the CF and SD behaviors. They each vary 24% from their real-world counterpart. However, their combined activation time falls within 0.2% of that of the original experiment. This difference can be attributed to the interaction between the CF behavior and the virtual environment. As mentioned in Section 4, the CF behavior tries to balance the change in energy between the channels of the sensor. This difference in energy over time comes about by the variation of reflections in each channel as the vehicle moves through the environment. In the real world, these variations occur due to shifts in reflections coming from smooth surfaces impinged by spatially continuous signals while the sensor moves through the environment. In simulation, reflections bounce off surfaces represented as meshes which are inherently segmented, and are impinged by a spatially discrete signal. This causes the accessible reflection paths to change more abruptly between movements, resulting in a higher rate of change over time, as thus more activation of the CF behavior.

## 5. SLAM

One important function of an autonomous system is to be able to operate in dynamic environments and adapt to current circumstances. Therefore, it is not enough to supply the system with a static map of the environment, which might not even be feasible to obtain for every setting. A solution to this issue is the use of a simultaneous localization and mapping algorithm. Using SLAM, it is possible to generate a map of an unknown environment and localize the vehicle within this map. Such a SLAM solution using pulse-echo radar has been developed in previous work [12]. It is a direct SLAM approach that uses the concatenated spectrograms of both sensor channels as a fingerprint for the current location, instead of performing feature detection as is common in many other solutions. The system estimates its position by dead-reckoning, e.g., path integration or visual odometry, and rectifies for any accumulated error by performing place recognition using the radar fingerprints. If a match between the current location fingerprint and a previous observation is made, the algorithm corrects for any potential pose error and perform graph relaxation to propagate the correction to previous poses. This solution is built on top of a SLAM implementation based on the biological neural processes relevant to self-localization in rats and bats [8,35]. In this section, we set out to replicate the results of previous work to assess the performance of the simulator for a SLAM use case [11]. For this purpose the vehicle was set to navigate the environment using its behavior-based controller. The measurements of the simulated radar sensor as well as the velocity command given by the controller are then used as input for the SLAM algorithm to create a topological map of the environment.

### Experimental Results

**Figure 7:**
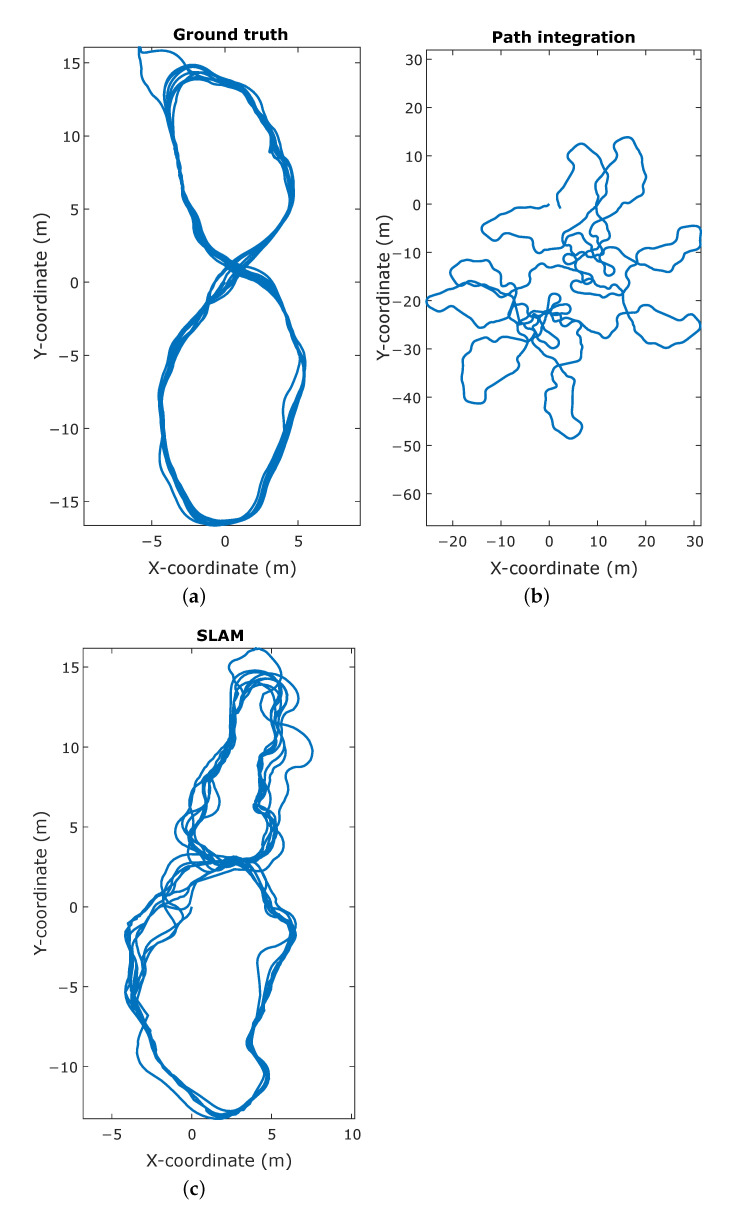
Comparison of the trajectories in the simultaneous localization and mapping experiment. (**a**) The ground truth vehicle trajectory, obtained directly from the simulation engine. (**b**) The trajectory as calculated by integrating the velocity commands given to the vehicle. (**c**) The trajectory reconstructed by the simultaneous mapping and localization (SLAM) algorithm, given the velocity inputs and simulated measurements.

**Figure 8:**
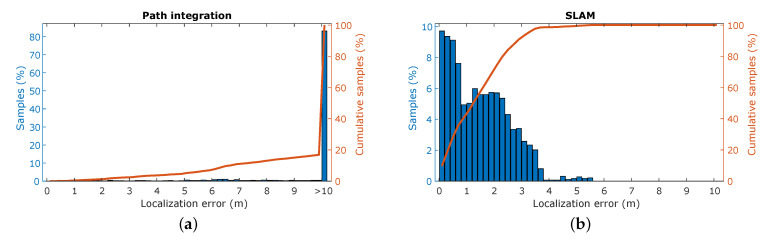
Simultaneous localization and mapping metrics. (**a**) The distribution of the positional error for the path integration compared to the ground truth. (**b**) The distribution of the positional error for the SLAM trajectory compared to the ground truth.

The resulting trajectory produced by SLAM shows a high resemblance to the ground truth trajectory, whereas the trajectory based on the path integration exhibits a large discrepancy, as illustrated in Figure 7. This is confirmed by the point-wise error of the trajectories compared to ground truth, as shown in Figure 8. For the path integration, 83% of the positional errors are larger than 10 m, while in the SLAM trajectory 95% of the errors are smaller than 3.2 m. This is a substantial improvement, and shows that the simulation is capable of producing a synthetic radar signal suitable for place recognition. However, in the real-world experiments performed in previous work, all positional error are below 1 m, meaning that there is still room for improvement to achieve completely realistic performance. This difference in performance might be attributed to several factors. First is again the discrete nature of the simulator. Small deviations in position can cause noticeable differences in reflection patterns, as explained in Section 4.2, which in turn impedes place recognition. Additionally, the lower stability of the controller, influenced by the same phenomenon, causes the vehicle to be subjected to higher jerk motions, causing the reflection patterns to vary even more over small time spans.

## 6. Design Space Exploration

**Figure 9:**
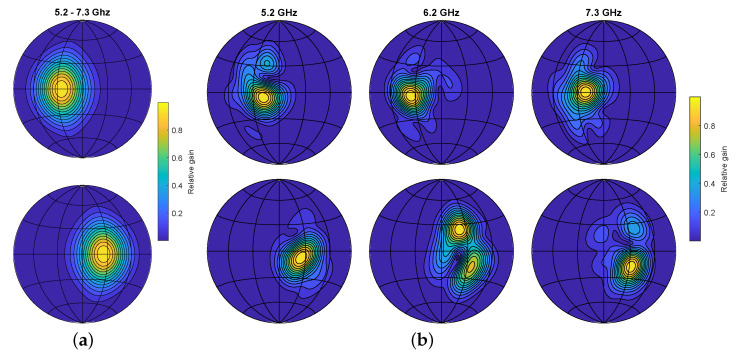
Lambert azimuthal equal-area projection of the echo-related transfer functions (ERTFs) used for the SLAM comparison. Top row represents the left channel, bottom row right channel. (**a**) ERTF with static lobe directivity. (**b**) ERTF with added spatiospectral variance

As mentioned earlier, the simulator can be used for design space exploration. To validate this, we set up an experiment to tests the difference in SLAM performance of particular antenna radiation patterns. For this purpose, synthetic ERTFs were generated by projecting spatial intensity functions onto a spherical surface. We presume that increased spatiospectral variance in a radiation pattern leads to better place recognition, as it does for bats [15,36]. This can be explained by the effect of an ERTF on an echo coming from a given direction. Looking at Figure 9, the ERTF with static lobes will apply the same filtering on the spectrum of an echo whether is it offset positively or negatively in elevation. The ERTF with spatiospectral variance, however, will apply a unique filtering pattern depending on the elevation. This results in a more unique fingerprint for the specific reflectors at a given location, and thus lowers the ambiguity in place recognition. In this experiment we compare two radiation patterns, the first of which features a stationary lobe in each channel with opposite offsets from the sensor heading;

(19) $$\begin{array}{cc} E_{L}\left(f,\theta ,\varphi \right) & =\exp(-\frac{1}{2}\;\cdot \;\frac{{\theta -\mu_{L}}^{2}}{\sigma }) \end{array}$$

(20) $$\begin{array}{cc} E_{R}\left(f,\theta ,\varphi \right) & =\exp(-\frac{1}{2}\;\cdot \;\frac{{\theta -\mu_{R}}^{2}}{\sigma }) \end{array}$$

with

$E_{L},E_{R}$: the ERTF components for the left and right channel respectively
θ: the azimuth angle
φ: the elevation angle
$\mu_{L},\mu_{R}$: the axis offset of the lobe
σ: the lobe width

In this case, $\mu_{L},\mu_{R}$ are −25° and 25° respectively. A frequency slice of the resulting ERTF is shown in Figure 9a.

Secondly, we apply a spatially varying function to the previously generated lobes. The spatial variation is generated by taking the inverse Fourier transform of a sparse matrix. By setting a select amount of elements to non-zero complex numbers, a pattern of superimposed sinusoids is generated, adding a random, yet continuous, characteristic to the radiation pattern.

(21) $$\begin{array}{cc} M_{L} & =F^{-1}\left(C_{R}\right) \end{array}$$

(22) $$\begin{array}{cc} M_{R} & =F^{-1}\left(C_{L}\right) \end{array}$$

(23) $$\begin{array}{cc} V_{L} & =E_{L}\;\cdot \;M_{L} \end{array}$$

(24) $$\begin{array}{cc} V_{R} & =E_{R}\;\cdot \;M_{R} \end{array}$$

with

$M_{L},M_{R}$: the spatial variation masks for the left and right channel respectively
$F^{-1}$: the inverse Fourier transform
$C_{L},C_{R}$: the coefficient matrices
$V_{L},V_{R}$: the spatially varying lobes

Here, $M_{L}$ and $M_{R}$ are 3D matrices with a dimension for the azimuth angle, elevation angle, and frequency. In this matrix we place 10 random complex coefficients at random indices below 5. Taking the inverse Fourier transform then generates a smoothly fluctuating spatial pattern. Multiplying the original lobed radiation pattern with this fluctuating mask gives the desired spatiospectral variance, as shown in Figure 9b.

**Figure 10:**
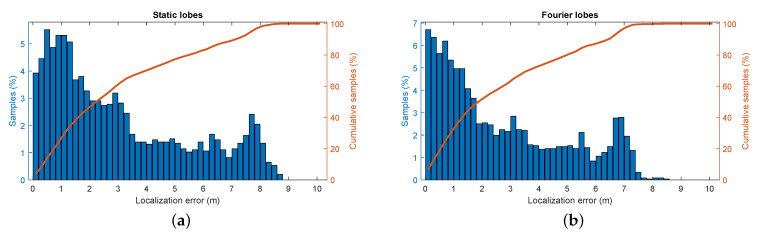
Simultaneous localization and mapping metrics for the different ERTFs. (**a**) The distribution of the positional error for the trajectory generated from the static ERTF compared to the ground truth. (**b**) The distribution of the positional error for the trajectory generated from the varying ERTF compared to the ground truth.

We again perform an experiment in which the robot is set to wander the environment. We then filter the incoming radar signals using each ERTF to obtain two sets of recorded signals and run the SLAM algorithm on both the data sets. The metrics of these experiments are shown in Figure 10. Here, a modest improvement in localization can be discerned when comparing the varying ERTF to the static-lobed ERTF. Additional metrics further confirm the hypothesis of improved place recognition by using an ERTF with increased spatial variability; the average error decreases by 11%, from 3.05 m to 2.70 m, and the 95th percentile localization error decreases from 7.70 m to 6.70 m.

## 7. Discussion

In this work, we presented a novel simulator for echolocation using pulse-echo radar. We analyzed the principles underlying the physical processes in both electromagnetic wave propagation and biological echolocation. We posit the idea that ray tracing can sufficiently approximate high-frequency signal propagation to be a useful tool in pulse-echo sensor design. The simulator was then implemented based on these processes, while employing optimizations specific to this application to maintain real-time performance. To validate this implementation, we replicated real-world experiments from previous work, allowing us to compare both results. In the autonomous navigation experiments, the outcome bears a strong resemblance to the original results. It can be classified as realistic behavior under the given circumstances. The vehicle moves on a stable trajectory through the environment, while activating the appropriate behaviors under the corresponding circumstances. A similar result can be seen in the SLAM experiment. Here, the algorithm was able to successfully localize the vehicle even for path integration exhibiting high levels of drift over time. The resulting topological map resembles the ground truth trajectory to a suitable degree. In addition to the realistic simulation of navigation and SLAM, the system also enables design space exploration of sensor parameters by allowing the user to modify most aspects of the sensor setup, such as the emitted signal, emitter and antenna layout, radiations patterns. We show experimentally that it is possible to improve the localization performance of the system by optimizing the spatiospectral variation in the ERTF, which can serve as an indicator of how to further develop the sensor hardware. In both replicated control and SLAM experiments, differences in performance were noted with respect to the real-world counterparts. We consider the main reason for these differences the fact the environment is spatially undersampled. Because of the segmented nature of mesh objects, a small displacement of the sensor might cause rays to strike a substantially different set of surface facets and thus produce a noticeably divergent set of reflection paths through the environment. Increasing the number of rays cast by the sensor would ensure a higher chance of rays traversing similar paths between time steps. This would in turn cause more stable echoes to be present in the signal, thus providing a more time-continuous character to the simulated measurements. We also maximized the potential of the simulator by basing it on the Unreal engine, which supplies a high amount of realism and control of the environment. On top of that there is a large library of structures, objects, and environments as well as vehicles available to replicate a wide variety of scenarios to test the sensor.

## 8. Materials and Methods

The simulation of the virtual environments, objects, world physics and ray-tracing were done in the Unreal Engine, version 4.22.3 [19]. Vehicle control and dynamics were provided by the Microsoft AirSim plugin, version 1.3.1 [16]. Point cloud processing and signal generation were performed in MATLAB 2020b using the following toolboxes: Signal Processing Toolbox, Statistics and Machine Learning Toolbox, Communications Toolbox, Aerospace Toolbox, Parallel Computing Toolbox.

## Data Availability

Data sharing not applicable. No in-depth statistical analysis on data sets was performed for this article. Experimental recordings and metrics are available upon request.
